# ON GESTATION AND MOTHERHOOD

**DOI:** 10.1093/medlaw/fwac030

**Published:** 2022-08-18

**Authors:** Zaina Mahmoud, Elizabeth Chloe Romanis

**Affiliations:** London Women’s Clinic, London, UK; School of Law, University of Exeter, Exeter, UK; Gender and Law at Durham, Durham Law School, Durham University, Durham, UK

**Keywords:** Gestation, Motherhood, Parenthood, Pregnancy, Reproduction

## Abstract

In English law, legal motherhood is allocated to the person who gestated. However, we argue that gestation—legally denoted as the “natural” source of parenting obligations—is often constructed as mothering, rather than the precursor to it. This means that women and pregnant people are treated as mothers *prior* to birth in legal and medical contexts. Since legal motherhood is an important status, defining the role an individual plays in a child’s life, the conflation of gestation and motherhood does not reflect that, legally, a fetus does not have personhood. This blurring between gestation and motherhood is metaphysically incoherent, as a fetus is not an entity that can be parented. This conflation poses a real harm to pregnant people’s autonomy, specifically those who do not intend to parent or who do not identify as women. More broadly, the medico-legal conflation of gestation and mothering is autonomy-limiting for all pregnant people as, resultantly, they may be coerced into obstetric intervention through legal processes. We argue for a better recognition of the differences between gestation and mothering, to promote autonomy and reflect the very different ways families may be formed.

## I. INTRODUCTION

One of the most immutable facts of English family law is *mater semper certa est* (the mother is always certain).^1^ The person who gestates and gives birth to a newborn is recognised as the legal mother of that child.^2^ Though established in Roman law, this principle remains at the root of English law today. It was emphasised by Lord Simon in the *Ampthill Peerage* case, in his statement that ‘[m]otherhood, although a legal relationship, is based on a fact, being proved demonstrably by parturition’.^3^ In England and Wales, therefore, legal motherhood is attributed solely based on the role played by a pregnant person in gestation. Law does not distinguish between mothering and motherhood—that is ‘between giving birth and being a mother’^4^; with this best exemplified by section 33(1) of the Human Fertilisation and Embryology Act (HFE Act) 2008 defining a mother as

the woman who *is carrying* or *has carried* a child as a result of the placing in her of an embryo or of sperm and eggs, *and no other woman*, is to be treated as the mother of the child.^5^

Equally, law does not differentiate adequately between pregnancy and motherhood, and it actively and inappropriately attributes parenting responsibilities to a pregnant person, as will be demonstrated in this article.^6^ Resultantly, law views a pregnant person *as if they already are a mother*.

In this article, we argue that law’s conflation of mothering and gestation is harmful, as it limits pregnant people’s autonomy, pressuring them to act in accordance with gendered norms associated with motherhood. In Section II, we explore the metaphysical incoherence of attributing motherhood to gestation and argue that a fetus cannot be parented. In this discussion, we provide the theoretical framework for our subsequent critique. Section III outlines motherhood as understood legally, analysing the production of the “natural” construction of motherhood emerging from gestation. Motherhood, we demonstrate, is taken as both biologically determined and gendered. “Mother” and “mothering”, as described by law, reiterate gestating as special and contribute to the construction of gestating as (a form of) mothering. We explain how this construction is metaphysically flawed, using our theoretical framework set out in the first section. In Section IV, we then illustrate the harms caused by law’s conceptual conflation of gestation with motherhood in medicine, due to the prominence of “good motherhood” narratives and maternal rhetoric in discussions about pregnancy. We focus on harms to surrogates, pregnant people making choices about their pregnancy and birth, and transgender people as legal parenthood is misaligned with their lived reality. In Section V, we outline suggestions for reform and further investigation.

## II. GESTATION IS NOT MOTHERING

The conceptualisation of pregnancy as the fetal container model—as ‘a unique state, involving as it does two distinct entities within one body’^7^—abounds in legal commentary. Despite contrary assertions, the fetus is *not* a distinct entity but is *part of* the pregnant person,^8^ due not only to geography, but also functional integration (the parthood model). This integration, as the reason why a person becomes a *pregnant* person, is precisely why the fetus must be understood as part of a person’s physiology, rather than as a distinct creature existing within them. The fetus is completely integrated into the pregnant person’s physicality, functionality, and physiology; any conceptualisation of it as separate and distinct is a socio-political act. Elsewhere, there has been persuasive defence of the parthood model, whereby the fetus is part of the pregnant person, like other parts of their anatomy.^9^ This metaphysical account of pregnancy is under-utilised in legal commentary, with the fetal container model often taken as a given.^10^ We adopt the parthood model to illustrate why a pregnant person cannot be described *accurately* as “mothering” the fetus during gestation.

By making no claims about the fetus’ (lack of) moral status, we accept the logical corollary of our arguments, that the fetus lacks any moral status. Expanding on Prabhpal Singh’s account of parental status, we argue that a fetus cannot be “parented”. Singh explains that ‘the fetus is not the sort of thing that can stand in a parent-child relation’, and that

The status of parent is relational status, to be a parent is not due to any intrinsic features or qualities of a person. Rather, to be a parent depends on having children of one’s own, and is therefore dependent on standing in relation with another, where that other is a child.^11^

### A. A Fetus is not an Entity that Can Be Parented

In their response to Singh’s relational account of parenthood, Bruce Blackshaw and Daniel Rodger argue that Singh does not coherently defend his claim of a fetus’ non-parentability.^12^ In what follows, we use Kingma’s account of the metaphysics of pregnancy (specifically adopting the parthood model) to defend Singh’s position that a fetus is not an entity that can be parented.

Contrary to widespread presentation of the fetus as free-floating, only anchored by the umbilical cord, the fetus is part of a pregnant person through its spatial integration within their anatomy.^13^ The fetus is ‘functionally and metabolically integrated, and interdependent with (other) parts’ of the pregnant person.^14^ Structurally, it is unclear where the fetus begins and the pregnant person ends, as it is ‘implanted within the uterine wall, within the maternal deciduous tissue, and is, at least in its early stages, completely covered by it’.^15^ Moreover, pregnant people often experience the fetus not ‘as a separate identity but as a “total bodily indwelling”’.^16^ A pregnant person’s physiology makes complex adjustments ensuring continued facilitation for fetal existence and controlling its internal environment.^17^

Although, as we argue, the fetus is part of the pregnant person, its future existence as a separate entity is continuously reiterated. The consistent socially—and medically reinforced—construction of the fetus as a ‘free-floating entity’^18^ downplays its complete integration within the pregnant person’s body. Importantly, such constructions do not dispel the reality of the fetus’ integration within the pregnant person. Claiming the fetus as distinct from the pregnant person because of its eventual separation from that person ‘conflates a possibility with its actualisation’.^19^ There is a lot, defended as morally significant,^20^ that must happen before the fetus is a separate entity—a baby. Meaningful differences exist between a fetus *in utero* and baby *ex utero*. Once born, it is a *distinct entity*. Ultimately, entities are not treated based on what they *might* be ‘but on what they are’.^21^

Following the parthood model, the fetus’ integration within the pregnant person results in an inability to describe a person as mothering while pregnant. Even when determined to ensure a successful pregnancy, when making decisions to ‘take care’ of their fetus, a pregnant person is taking care of *themselves* in respect of their future interest in becoming a parent.^22^ Parenting is a relational activity: however, one can only interact with the pregnant person and not the fetus. Even ultrasounds, which provide a ‘view to the fetus’, require physical contact with the pregnant person. During gestation, the fetus ‘is completely mediated by the material body’ of the pregnant person.^23^

### B. Pregnancy and the Relational Status of Parenting

In *Re G*, Lady Hale recognised that

the process of carrying a child and giving him birth … brings with it, in the vast majority of cases, a very special relationship between mother and child, a relationship which is different from any other.^24^

Regardless of any affiliation with the fetus, the relationship between a pregnant person and a fetus *is* distinguishble from parenting relationships,^25^ because the entity born alive—the baby—possesses the quality of ‘natality’ once it is no longer part of the pregnant person.^26^ It enters the world as an ‘inescapably situated’ being,^27^ dependent on others. Through their natality, babies—distinct entities physically existing amongst others—are ‘vulnerable in various ways. We begin life helpless and vulnerable … As relational beings, we are vulnerable to being damaged by failures of care’.^28^ Through their dependency, babies are capable of interacting with and experiencing physical contact with others. Babies require feeding, shelter, warmth, and affection, with these basic human needs attended to by those acting to protect and care for them. Parents are recognised as those responsible for meeting such needs and are obliged to do so to avoid criminal liability for neglect.^29^

Helen Watt claims that ‘pregnancy is maternal—it involves basic maternal support for a dependent child’.^30^ We disagree. Before birth, an individual performs gestational labour. Sustaining a pregnancy involves significant sacrifices: e.g., sharing bodily resources and facing socio-cultural expectations associated with visible pregnancies. However, performing this labour is not *interactional*, but is an *internal* physiological demand. Gestational labour cannot be shared—unlike parental care labour. After birth, a baby is supported differently. It is no longer integrated within the pregnant person’s body, it no longer exists in an intangible domain: a baby can be touched, smelled, heard, and held. Thus, any characterisation of gestational labour, where the fetus is integrated within the pregnant person, as equivalent to maternal care provided after birth is misplaced.

Contesting Singh’s claim that a fetus cannot be the subject of “parenting” labour, Blackshaw and Rodger argue that

The parent–child relation is … a continuation of an existing relationship formed during gestation … it is important to recognise that the maternal–child relationship begins during the normal process of gestation. Maternal–fetal attachment is a well-documented phenomenon … Birth marks a significant change to the parent–child relation, but it is implausible to claim it originates with birth in the case of the mother.^31^

Yet, the bond described is not a *relationship* but, we argue, a *physical integration*: the fetus and pregnant person are ‘flesh-and-blood bonded’.^32^ A pregnant person and fetus share the placenta—a new organ—formed of tissues from both,^33^ and genetic material following naturally occurring microchimerism.^34^ Pregnancy *is*, thus, a unique experience, and it is the pregnant person’s experience. Any relationship between the pregnant person and the fetus is *not* akin to one with a newborn. Gestation is a generative, creative process. Parenting, while also a creative endeavour in the making of an individual,^35^ involves a *proactive* two-way interaction of *two separate* entities, and ‘the physical gestation of a child is … neither necessary nor sufficient for the development of a loving parental bond’.^36^ This is evidenced by surrogates who do not see themselves as “parenting”.^37^

While we recognise that many pregnant people intending to parent value their fetus *qua* potential child, as discussed later in this subsection, this may be acknowledged without negating the metaphysical fact that a fetus is not a separate *parentable* entity. Indeed, we do not deny the importance accorded to a fetus by those intending to parent, but we note the dangers and inaccuracies of describing this importance through the same social scripts used for parent–baby relationships. As we argue later in Section IV, this distinction between fetus and baby cannot be legally blurred and must be maintained not just to recognise metaphysical realities, but also to prevent the erosion of pregnant people’s autonomy and identity.

Often, pregnant people are described as “pregnant mother” or “mother” in academic literature. Some argue that since ‘pregnant women often refer to themselves as mothers—attributing the term “mother” to a pregnant woman ‘hardly seems inappropriate—and a mother is a parent by definition’, with this evidencing the existence of a pre-birth parenting relationship.^38^ However, as Lachlan de Crepigny and others have noted correctly, it is simply ‘grammatically incorrect to use the word *mother*’ to describe a pregnant person^39^ because of the different responsibilities. Moreover, such literature describing pregnant people as “mothers” is criticised for its determinism.^40^ This determinism is especially inappropriate in descriptions of pregnant people seeking abortions as mothers to the fetuses they carry. Although many undertaking a wanted pregnancy may consider themselves already to be mothers while pregnant, this is not a universal experience, as shown by the experiences of some surrogates,^41^ and pregnant people identifying as non-binary or trans/masculine.^42^ In fact, a pregnant person’s understanding of their pregnancy, their body, and the fetus is entirely subjective. Even where gestation is considered by those undertaking it as care labour, it is conceptualised as ‘getting to know the fetus’, and so remains different from interacting with one’s baby because of the lack of reciprocity.^43^ Gestation is the transcendence of self and embodiment of caring, rather than interacting with and rearing another entity.

We have argued that the metaphysical facts and ordinary use of the term “mother” inaccurately depict gestation. Later, in Section III, we explain that referring to pregnant people as mothers is actively harmful, as it attempts to ‘*force* thinking about “pregnancy as parenting” on a pregnant person’.^44^

### C. Significance for Legal Parenthood

Law assigning legal motherhood unquestioningly to the gestating individual before birth is odd, as gestation is *not* mothering. This gestating individual—who has not yet parented—is automatically vested with parental responsibility,^45^ despite gestation and parenting being different forms of labour. This legally codified and socio-culturally reinforced assumption is so embedded that abdication of automatically afforded legal motherhood status and associated parental responsibilities, in England and Wales, is a complex, time-consuming legal process, as demonstrated by the processes obtaining a parental order, or adoption.^46^

Yet, changing societal attitudes to parenthood and the increased use of assisted reproductive technologies (ART) have contributed to increased academic interrogation of how legal motherhood is automatically attributed. For example, Kirsty Horsey notes that in the context of surrogacy, ‘law singularly fails to reflect … lived experience: the view of surrogates that they are not mothers’.^47^ Julie McCandless highlights how recording of vital events, like birth, ‘will inevitably flatten subjectivities and the richness of an individual’s personal narrative’.^48^ Currently, surrogates’ experiences are not recognised in law^49^: they do not view their undertaking of gestational labour as an act of mothering, nor do they see themselves as mothers, nor—importantly—do they intend to mother. Yet, they are recognised as legal mothers solely on the basis of their gestational labour. While this experience of surrogacy is, of course, not universal, British empirical data support this conclusion.^50^ A contrary argument, that surrogates are mothers, makes little sense when the distinction between the metaphysical realities of pregnancy and “mothering” is afforded due attention.

Presuming the pregnant person intends to mother the resulting baby is a relic of a time when it was biologically impossible and socially unacceptable for people not to “mother” their biological children, largely due to women not being recognised as equals, and being expected to bear children and raise families. Despite reinforcement through conservative societal ideals, this view is not as ingrained in society as it once was. There is increasing societal acceptance of diverse family forms. While it is important to highlight that there is not yet sufficient support for trans individuals who want to become parents, nor is law or policy good enough at supporting diverse family formation, some changes have enabled same-sex couples to build their families. Same-sex couples can receive funding for fertility treatment in some parts of the country,^51^ they can participate in surrogacy arrangements, female same-sex couples can both be recognised as the legal parents of a child conceived through ART at birth,^52^ and they can adopt.^53^ Law’s persistence that the pregnant person *is* already the mother additionally fails to recognise this diversity in family formation and ignores the autonomy of people with the physiology to carry a pregnancy. This results in systematic discrimination against women and pregnant people seeking to make autonomous choices about their obstetric care, as we discuss in Section IV. Before this, we use our established theoretical framework to interrogate and critique how law specifically contributes to the construction of gestating as mothering.at birth

## III. MOTHERHOOD AND LAW

Legal parenthood is an indivisible and exclusive status; a child can only have a maximum of two legal parents in England and Wales.^54^ As a legal construct, parenthood recognises an ongoing relational status and recognises the individual responsible for raising the child.^55^ Although some people do socially “parent” without formal recognition of their parental status,^56^ the importance of legal parenthood cannot be understated. Legal parenthood allows ‘real and effective rather than theoretical and illusory’ protection of associated rights and responsibilities.^57^ A legal parent with parental responsibility is legally responsible for the child and their welfare,^58^ and has ‘standing’ to enforce their child’s legal rights.^59^ Legal parenthood, in its symbolic sense and its practical implications, is clearly a relational status. A “parent” is defined in relation to their (presumed) role in a child’s life. While legal motherhood as a status is distinct from the act of “mothering”, which is the role a person plays in a child’s life,^60^ the way law is written describes “motherhood” in a way that simultaneously denotes mothering.

A fetus is not a legal person and only gains legal personhood at birth,^61^ when it is alive and has been delivered completely separated from the pregnant person’s body.^62^ A fetus, thus, *cannot* have a legal parent as it is not a legally recognised entity capable of being the subject of ‘parenthood’. By virtue of not being a legal person, a fetus neither has legal rights nor legal standing. No legal claims can be made by others on its behalf; for example, to protect its welfare.^63^ Legal parenthood *cannot* apply to it; no one can *be* its parent. However, significantly, the *mater est* principle as codified in the statute regulating ART specifies that a woman who ‘*is carrying or has carried* a child… is to be treated as the mother’.^64^

In this section, we offer four critical observations about the rules surrounding legal motherhood in light of the theoretical framework we have outlined.

### A. Biological Determinism

Legal motherhood remains rooted in a particular biological role and process, unlike the legal status of ‘father’ or ‘second female parent’ in the case of same-sex female couples.^65^ Legal motherhood emerges from a perceived ‘natural’ source, one that is grounded in gestation. Law does not define fathers or second female parents based on biological contributions^66^; yet mothers are recognised through a particular biological contribution. This feeds into patriarchal notions of the female body’s function and role as being biologically determined. Although experienced primarily by women, motherhood is, in fact, ‘defined, controlled, and given legal content by patriarchal ideology’.^67^ Law’s understanding of ‘women, women’s nature, women’s capacities, and women’s experiences—women refracted through the male eye’ shapes its construction of motherhood.^68^ Law reinforces shared notions of what is appropriate behaviour for legal mothers^69^; in what follows we consider these standards and their embeddedness in law.

At birth, legal parents are necessarily mothers, and if married/in a civil partnership, their spouse/partner is afforded the legal status of father or second female parent, each defined differently. While the latter two are afforded some degree of choice about their legal status, biological determination limits pregnant people’s freedom to make similar arrangements about their legal motherhood—or fatherhood, in the case of trans/masculine people. Legal fatherhood and second female parenthood are much more easily rebuttable since their legal recognition emerges from that person’s particular *relationship to the gestating individual* and not to any specific role played in conception. Legal fatherhood is attributed to the legal mother’s husband/civil partner, regardless of any shared biological connection with the child.^70^ Alternatively, the legal father is the man consenting to this legal status during ART provision where the woman receiving treatment consents to his legal fatherhood.^71^ Alternatively still, the legal father is the man registered on the birth certificate.^72^ Similarly, second female parenthood arises either through marriage/civil partnership with the legal mother, regardless of any shared biological connection with the child,^73^ or the woman who consents to being treated as the second female parent during ART provision where her unmarried partner receiving treatment consents as well.

Legal mothers have automatic parental responsibility, whereas their unmarried partners only obtain parental responsibility following their consent, when named on the birth certificate^74^ or through other legal mechanisms that involve petitioning the courts. Unmarried fathers and second female parents may be entered on the birth register, though only ‘with the co-operation of the child’s mother or where there is a court finding of his paternity’,^75^ thereby necessitating the *gestating person’*s involvement.

As we have demonstrated, neither legal fatherhood nor second female parenthood is based on biology—yet legal motherhood remains rooted in biology. Only legal motherhood, which is perceived as *innately* existing within a particular individual, is *truly* irrebuttable. This status can only be abdicated through a limited number of proscribed methods—adoption or parental orders. Importantly, once legal motherhood is abdicated, that child has no legal mother. For example, following surrogacy, once a parental order is granted, the surrogate’s legal motherhood is extinguished, as is her spouse’s legal fatherhood/second female parenthood, where relevant, or one of the intended parents’ legal fatherhood/second female parenthood, where relevant.^76^ The intended parents are vested with legal parenthood, but their legal status is not as legal mother(s) or legal father(s), but rather as Parent 1 and Parent 2.

In this way, law affords those who do not gestate and birth freedom to make social determinations about their legal parental status, whereas biological determinism inhibits the freedom of gestating people to make such arrangements. As Pam Lowe explains, since (primarily) women are capable of gestating, ‘ideas about natural motherhood and womanhood are intertwined’.^77^ People with female-assigned physiology were and continue to be defined as different due to their unique child-bearing capacity.^78^ This has played a part in females being seen as distinct from—if not inferior to—males,^79^ which is essentially a form of sex-based discrimination. Automatic assignment of legal motherhood to those who give birth reinforces patriarchal notions about gender roles, because through sustaining a pregnancy, people with female-assigned physiology are legally tied into rearing responsibilities after birth.^80^ Such presumptions emerge from legal motherhood because “mother” is legally and socially understood as a relational status, describing a person’s standing in relation to the care provided to a child. Once a mother, society defines that person through this status for life. Their identity as an autonomous person is lost and is replaced by a socially acceptable identity as a mother.^81^ Before the emancipation of women, as well as the development and accessibility of effective contraception for female-assigned physiology, a woman’s natural duty was bearing children.^82^ All women were, and continue to be, considered potential mothers.^83^

This conceptualisation of legal motherhood binds and confines women to a biological destiny, assuming that caring responsibilities after birth innately accompany gestation. This perpetuates the notion that it is ideal for the baby—and for the gestating person—for this person who gave birth to be their carer.^84^ Within the context of gestation, legal rules clearly have normative force in conscripting the female body.^85^ Currently, legal motherhood fails to reflect the diversity of family formation, e.g., where the gestating person does not intend to mother after birth, such as surrogacy and adoption.^86^

Furthermore, that legal mothers cannot abdicate parental responsibility by giving ‘effective consent’ to adoption or parental orders until six weeks after birth^87^ illuminates how law constructs the person who gestates and gives birth. Law reinforces pregnancy as innately inhibiting a person’s decision-making abilities, due to hormonally induced unpredictability, a further patriarchal assumption of people with female-assigned physiology being ruled by their wombs.^88^ Law simply does not entertain ‘the idea that a [person], after giving birth, might make a *rational* decision not to become a mother’.^89^

### B. The Perpetuation of Gender Norms

Concerns related to “certainty” of parentage partially explain the historical presumption of gestation as determinative of motherhood. Prior to the development of ART, when a person gave birth, their biological relation to the baby was certain. Paternal certainty, however, was not. Indeed, often women were treated with suspicion as to the true paternity of their child—especially where they were unmarried.^90^ As noted in the *Ampthill Peerage* case,

A woman can have sexual intercourse with a number of men any of whom may be the father of her child; though it is true that modern serology can sometimes enable the presumption to be rebutted as regards some of these men (…) since fatherhood is not factually demonstrable by parturition, it is questionable.^91^

The proliferation of ART, widespread direct-to-consumer genetic testing, and the depletion of the social importance of ‘legitimacy’ (fuelled partly by legal reforms)^92^ resulted in the social demotion of ‘certainty’ concerns. However, social rules remain conscripted around these suspicions of gestating people (usually women), and the legal mother is generally required to declare a child’s paternity,^93^ where relevant, subject to some exceptions.^94^ Under the Births and Deaths Registration Act 1953, as amended, an unmarried *gestating person*, as the legal ‘mother of the child’ is obliged to register a birth, with the Act stipulating that ‘no person shall as father of the child be *required* to give information concerning the birth of the child’ (emphasis added).^95^ A legal father/second female parent’s ability to register the birth is contingent on their relationship to the legal mother, either through marriage/civil partnership. Although there are policy justifications for this requirement,^96^ the different weighting of legal mothers’ responsibilities at birth, compared to fathers and second legal parents, is clear.^97^ While recently having only the legal mother named on the birth certificate does not result in significant scrutiny,^98^ law as written, imbues *single* legal mothers with the responsibility of registering and declaring paternity.^99^

Clearly, a matrifocal approach to legal parenthood has been adopted in England and Wales. Historically, in order to ensure legitimacy, the marital presumption recognises the gestating person’s husband as the legal father (*pater est quem nuptiae demonstrant*).^100^ Until recently, gendered assumptions underpinning legal motherhood were never fully discussed. Yet, the gendered construct of “mother” presumes the existence of a complementary “father”, and in same-sex parenthood, both partners are socially considered mothers (or fathers) even where they themselves reject gendered terms, preferring ‘parent’. As highlighted in the previous sub-section, with same-sex parents, the non-gestating partner is legally the ‘second female parent’,^101^ and their legal parenthood is akin to legal fatherhood and so is derived from their relationship with the legal mother.^102^ Importantly, the reluctance to recognise a second legal mother reveals this individual’s role as ‘an additional, somewhat ill-defined, parenting presence’,^103^ less than a mother—or even a father. Their legally recognised parental status *as* ‘second female parent’ reiterates gestation as central to legal motherhood.

Indeed, in *R (on the application of McConnell)*,^104^ the Court of Appeal, affirming an earlier High Court decision,^105^ declared the legal mother as the person undertaking gestation *irrespective of gender identity*. Acknowledging that Parliament had not completely “de-coupled” the concept of “mother” from gender, the High Court ultimately held that legal motherhood reflected ‘common sense, common experience and the basic facts of life’.^106^ Such a statement obscures how “common-sense” assumptions are grounded in gendered, hetero- and cis-normative stereotypes. The Court of Appeal similarly neglected this reality, reiterated that giving birth resulted in automatic parental responsibility, and recognised a ‘material difference between a person’s gender and their status as a parent’.^107^ Parliament gave “parent”—and “mother” and “father”—distinct meanings, each fulfilling different but complementary legal roles, and the Court stipulated that “mother” could not be replaced with “parent” as this would ‘amount to judicial legislation’.^108^ This is seemingly notwithstanding the fact that “parent”, “mother”, and “father” are often used interchangeably and are ill-defined within legislation.^109^ Both *McConnell* judgments emphasised gestation as the ‘essence’ of legal motherhood.^110^

The decisions in *McConnell* demonstrate ‘the curious reinforcement of gender in reiterating a link between female biology and a term that remains gendered in its popular use’.^111^ This conclusion is further strengthened by the unexpected determination that

The fact that a person’s gender has become the acquired gender under [the Gender Recognition Act 2004] does not affect the status of the person as the father or mother of a child.^112^

This interpretation of the Gender Recognition Act 2004 did not correspond with existing academic opinion,^113^ and clearly reiterates gendered biological determinism around reproduction.^114^ This judgment illustrates how law reproduces and perpetuates patriarchal relations, namely family and gender divisions,^115^ regardless of (mis)alignment between legal discourse and lived experiences. Law and its specific language are social conditioning tools because of their ‘expressive effect’.^116^ Legal language is able to encode people’s relationships with meaning; words with so much formal recognition describing individuals and their behaviour, and intending to regulate conduct, simultaneously create meaning. In this way, legal language reproduces power structures. Formally labelling individuals as fulfilling particular roles is powerful. The decisions in *McConnell* aptly revealed the fact that legal terms are autonomous from social understandings and lived reality.

The High Court in *McConnell* reiterated the fact that naming the person who gave birth on the birth certificate as the legal mother serves a child’s best interests, despite the misalignment with the lived reality of the situation. Automatic recognition as the legal mother was deemed necessary for a coherent birth registration scheme and consistent records,^117^ but the rationale for prioritising consistent recordkeeping over reflecting the lived reality was not explained.^118^ Historically concerned with private property rights,^119^ birth certificates have long been ascribed an inordinate amount of importance. Clear birth records were deemed central in *McConnell* as a matter of ensuring accurate origins, though historically, these records were crucial with regards to legitimacy and inheritance. Some may view them as true records of genetic origins—not ‘deliberate lies’—^120^notwithstanding the reality that gamete donors and intended parent(s) in surrogacy arrangements are not named. Furthermore, neither legal motherhood nor second female parenthood (for same-sex parents) is ascribed based on genetics. Thus, the current birth certificate scheme further reinforces legal motherhood as emphatically grounded in gestation.^121^

### C. Mothering as ‘conscription’

Consideration of the social position of fathers reveals that they are—in some ways—perceived as volunteers, while mothers are conscriptees.^122^ This is seen through the praise provided to fathers providing day-to-day caring for *their* children or how spending quality time with *their* children without the mother results in praise for ‘babysitting’.^123^ Furthermore, the lasting social implications of abdicating legal parenthood are more drastic for legal mothers than for legal fathers or second female parents. There would be ‘widespread alarm’ were mothers to abandon children on the same scale as the (somewhat normalised) absence of fathers.^124^ The difference in the degree of socially afforded responsibility is reinforced by legal consequences, most obviously in how identifiable law requires the legal mother to be, and how severe the consequences are when a mother fails to act “like a mother” in “mothering”’ after birth. For example, concealing a birth where the baby (capable of being born alive) does not survive is a criminal offence.^125^ Even where the baby survives, a formerly pregnant person commits an offence if they:

unlawfully abandon or expose any child, being under the age of two years, whereby the life of such child will be endangered, or the health of such child have been or shall likely to be permanently injured.^126^

Primarily, this offence of concealment has been used ‘in circumstances where [women] had been suspected of being responsible for the death of their infants’.^127^ While homicide is difficult to prove, concealing a birth is not. Deployment of this offence where the death of a baby following birth is not reported results in the behaviour of the gestating person being ‘assessed next to ideals of motherhood’, with prosecutors’ narratives painting them as ‘rejecting motherhood’ based on ‘unmotherly’ behaviour.^128^ Concealment, abandonment, and homicide all carry custodial sentences, but concealment and abandonment are much easier to prove. Concealment is very rarely prosecuted; that concealment remains on the statute books reinforces normative suppositions about the behaviour of people who birth as “failed mothers”. The existence of the offence assumes its necessity in preventing “poor mothering” from harming babies after birth.

If a gestating person fails to abide by societal expectations on birthing supervised and reporting a stillbirth, for example, they are treated with suspicion and punished, effectively for failing as *mothers*. The offence of ‘abandonment or exposure’ reiterates the same expectations, notwithstanding the offence’s gender-neutral construction, whereby a person can be guilty even if they did not gestate. This offence is normally used to charge women who have “abandoned children”. The construction of the offence enables the criminalisation of a person who has birthed leaving a newborn in an objectively safe place (e.g., a hospital), since there is no requirement that the baby is harmed—only that their life is endangered—which is arguably whenever a baby is left unattended anywhere. Despite the offence centring endangerment, its construction demonstrates how the offence is not *wholly* about the baby’s safety, but instead the importance of a legal mother’s identifiability, suggesting the presence of an innate wrong if they fail to identify themselves.

The offences of concealment and of abandonment are just two examples of how law is underpinned by ideals of motherhood and placing specific on pregnant and birthing people to prioritise fetal welfare over their own.^129^ More widely, legal motherhood *qua* construct is underpinned and supervised by patriarchal ideology,^130^ which epitomises altruism, as can be seen in judicial descriptions of mothers as ‘inherently nurturing and loving toward their children’.^131^ Traditionally, good mothers are supposed to be self-sacrificial and selflessly available to their children, the ‘divinely appointed guardians of the family’.^132^ Seemingly reflective of altruism, gestation is significantly weighted above all else in law, with ‘the fetus [taken] to be a part of the mother until it has an existence independent of the mother’.^133^

Despite recognising the fetus as *part* of—not separate from—the gestating individual, legal motherhood remains indivisible between gestating (and giving birth) and raising the baby.^134^ This is seen in the codified definition of legal motherhood, section 33(1) of the HFE Act 2008, discussed in the next sub-section. Recognising and reflecting a person’s unique role in pregnancy and birth in *Re G*, Baroness Hale drew attention to the significant physiological—and oftentimes, emotional, and psychological—investment in carrying a fetus and undergoing labour and delivery.^135^ Despite also noting the importance of the social institution of mothering, the focus placed on a maternal tie based on presumed *in utero* bonding still reiterates the idea that the child’s best interests would be served, prima facie, by the gestating individual mothering them, regardless of the circumstances. In this way, law reinforces the view that ‘naturally’ the gestating person is—and wants to be—the caregiver.

The ‘tender years’ doctrine, holding that a mother’s care is ordinarily in the child’s best interests, was significant for maternal rights in the 19th century,^136^ but has since whittled away. This was partly due to the enactment of the Children Act 1989, which in asserting that a child’s ‘welfare shall be the court’s paramount consideration’^137^ somewhat did away with the presumption that their best interests are served by the legal mother *by default*. The Act provided other parties the opportunity to make the case that they better serve the child’s welfare. Notwithstanding this, the person giving birth acting as the mother still remains the starting point for assessing a child’s best interests.^138^ Consequentially, while waiting for the granting of a parental order or formalisation of adoption, those not intending to mother could feel intense pressure to mother in the interim. Practically, they must “perform” as mother and undertake the formal tasks required of the person with parental responsibility, as they automatically have that responsibility.^139^ For example, if medical treatment is required immediately or shortly after birth, the person who gave birth is legally responsible and must consent to this treatment. Consequently, the legal mother may be the only person who can consent to something as simple as the administration of a routine painkiller.

### D. Gestation qua Motherhood?

Our final observation about the construction of legal motherhood is its specific conflation of gestation and mothering. A fetus does not have legal personhood and is thus not afforded the rights and protections of a child.^140^ This recognition is important in order to protect pregnant people’s rights.^141^ While babies can be made wards of a court when it is necessary to protect their welfare, in *Re*  *F*, the Court of Appeal refused an application to make a fetus a ward of court.^142^ The Court held that it could not ‘consider with any equanimity’ extending protections designed to promote children’s welfare to fetuses, because this would force pregnant people to forego fundamental freedoms.^143^ Legal recognition of a fetus as a child would render a pregnant person ‘a fetal container’,^144^ in such a manner that judgments in several arenas have refused to find is the case.^145^ Notwithstanding their lack of legal personhood, the construction of a pregnant person as a mother affords, we suggest, legal protection to fetuses. The conflation of gestation and mothering resulting in this legal protection of fetuses proceeds indirectly, via social expectations—legally supported—placed on pregnant people to act “like mothers”. These expectations are reinforced in legal language, social norms, and medical norms, as we show in Section IV.

Baroness Hale’s description of a pregnant person’s unique ‘contribution to the welfare of the child’ emerging from the special relationship formed during pregnancy^146^ exemplifies how gestation and mothering have collapsed onto each other within legal discourse. Pregnancy is described here as effectively facilitating a mothering relationship, echoing Blackshaw and Rodgers’ claim of this relationship pre-dating birth.^147^ In legislation, pregnant people are consistently described as mothers, with no distinction drawn between gestation and post-birth, best evidenced by section 33(1) of the HFE Act 2008: ‘the woman who *is carrying* or *has carried a child* […] is to be treated as the mother of the child’.^148^ Similar to our discussion in Section II on the differences between a fetus *in utero* and a baby *ex utero*, this wording conflates the person’s legal status while pregnant with that afforded to them at birth as a description of their relationship. Legal motherhood cannot be recognised until birth, since, as we have shown in Section II, parenthood is a relational status. Any recognition of (legal) motherhood prior to birth is legally and metaphysically incoherent.

More recently, rather than use the standard gender-neutral drafting—“pregnant people”—which has been in place since 2007—^149^the 2021 Ministerial and other Maternity Allowances Act was specifically rewritten to include “mother” and “expectant mother”, defended as ‘legally acceptable and more inclusive than other suggested alternatives’.^150^ Even in legislative debates on egg freezing, where those receiving this treatment have not even become pregnant, let alone “parented”, these individuals were referred to prematurely as ‘mothers’.^151^ Similarly, in case law, pregnant people are referred to as “mothers” or “expectant mothers” in situations where we suggest this is inappropriate; we explore this further in the following section. In sum, when pregnant people are inappropriately described in law as mothers *prior* to birth, they are subject to regulatory frameworks with significant normative implications for their treatment and behaviour.

## IV. LEGAL AND MEDICAL CONCEPTUALISATIONS OF GESTATION AS MOTHERING

In the previous sections, we have demonstrated the flawed assumption that mothering flows from—and begins within—gestation. Notwithstanding this incoherence, in both law and medicine, the conceptual confusion implying gestation *is* mothering persists. Crucially, as the fetus is increasingly more visible through technology, the pregnant person and the fetus are constructed as *separate*. Medical technologies afforded healthcare providers a view of—and into—pregnancy and fetal development, thereby engendering profound changes in the medical treatment of pregnancy, with increased responsibilities placed onto the pregnant person.^152^ ‘Floating free, attached only by the umbilical cord’,^153^ the fetus is rendered autonomous, despite this construction being at odds with reality, as the fetus is a part of the pregnant person.^154^ This blurred distinction between fetus *in utero* and baby *ex utero* contributes to and reinforces the (conceptually unsound) construction of the pregnant person as a mother.

This blurred distinction is seen in clinical practice guidelines, which aid physicians in patient management and should employ precise terminology. Yet, the latest National Institute for Health and Clinical Excellence (NICE) guidance refers to pregnant people as ‘women’ or ‘mothers’, and notes that these terms ‘should be taken to include people who do not identify as women but are pregnant or have given birth’.^155^ Use of the term ‘mother’ is conspicuously absent in abortion care guidance—with the exception of one instance—^156^ perhaps indicating that neither the pregnant person nor the physician views the fetus as a patient in these situations. By contrast, the British Medical Association (BMA) guidance recommends using gender-inclusive language—‘pregnant people’ and not ‘expectant mothers’.^157^ While this is welcome, as we similarly value inclusivity, the focus on inclusivity does not address how the term “mother” is only appropriate after birth (when there is an entity to parent), *and* where the birthing person intends to parent *and* identifies as a ‘mother’. Without also considering the harm of the implication that pregnancy is mothering in the language used, the expectation of self-sacrificial behaviour—as we will demonstrate—continues to limit the autonomy of pregnant people of all genders.

In many clinical settings, healthcare providers frequently continue to refer to pregnant people as ‘mum’ or ‘mother’.^158^ Furthermore, authors of articles in academic medical journals often use the term “pregnant mothers”, and although some have argued that this ‘hardly seems inappropriate’, given the fact that most pregnant people intend to parent,^159^ this rhetorical choice reinforces gestation *qua* motherhood. Such language presupposes the pregnant person’s intention and identity, subjecting them to an autonomy-limiting regulatory framework of medical and moral discourses wherein they are ‘supposed to engage with surveillance medicine, even if some are able to reject particular tests’.^160^

Use of maternal terms such as “mum” or “mother” in clinical contexts presumes that there is or has been a decision to sustain a pregnancy *and* to *keep and rear* the resulting baby as a mother. Healthcare providers’ language can influence pregnant people’s decisions and choices, with important consequences. In the context of abortion,^161^ referring to a pregnant person as “mother” and the fetus as “baby” may impact a person’s reproductive autonomy. Specifically, this language would make an abortion-seeker or a person experiencing a miscarriage uncomfortable, through its implication of *parental* responsibility for an entity that they may not recognise. For this reason, experienced abortion providers do not use such terms. This maternal language is more likely in obstetric care provision, and might be particularly difficult in the context of wanted pregnancies where fatal congenital abnormalities become apparent.^162^ As de Crespigny and others explain, language can be very powerful and there could be serious long-term psychological consequences for persons who choose to terminate when clinicians use charged language such as ‘your baby’ or ‘your child’.^163^ Beyond abortion, gestation’s conflation with mothering allows constant scrutiny of a pregnant person’s decisions.

From the outset of pregnancy, a pregnant person is viewed *as* a mother, the ‘maternal status and obligation start with the zygote’.^164^ Early on, a pregnant person is directed to adopt a ‘fetus-first’ mentality, prioritising the fetus’ needs over their own. Given the societal investment in people’s pregnancies, pregnant people’s behaviour is publicly scrutinised. While pregnant, they are expected to demonstrate commitment to the fetus, and restrict their lifestyle as needed; a pregnant person is a “bad mother” when acting in ways potentially harmful to the fetus. Pregnant people face considerable pressure to make the “right” choices about their pregnancy in a way that prioritises the fetus and its development, with their own health and wellbeing considered secondarily. These choices involve deference to accepted medical understandings of responsibility, and specifically minimising any potential harm to the fetus.^165^

Increased knowledge about the physiology of pregnancy, is accompanied by greater emphasis on controlling pregnancy. Pregnant people failing to act “maternally” are held responsible for endangering the fetus or potential adverse outcomes. Consequently, pregnant people are subject to public scrutiny and social control by healthcare providers. As Elizabeth Armstrong notes

The perception of an increasing threat to the fetus has enabled doctors to extend their monitoring and control of pregnancy and birth beyond the examination room, beyond the labor and delivery room, into their patients’ private lives.^166^

Where pregnant people make ‘irresponsible’ decisions, they are ‘bad mothers’ by *potentially* putting the fetus at risk.^167^ As a fetus does not have legal standing, a pregnant person is neither criminally nor civilly liable for damage done to the fetus through treatment refusal or ‘risky’ behaviour.^168^ In *Attorney-General’s Reference (No 3 of 1994)*,^169^ the House of Lords held that a fetus’ death *in utero* was not murder, but if a baby was born alive and subsequently died of injuries sustained *in utero*, a homicide conviction was possible with clear proof of transferred malice.^170^ The judgment does not explicitly mention pregnant people, and thus does not exclude them. Therefore, there remains, technically, the possibility of a prosecution for gross negligence manslaughter,^171^ or unlawful act manslaughter, if it can be shown that a pregnant person acted in grossly negligent or unlawful manner while pregnant causing their baby to die after being born alive. There has, however, never been such a prosecution testing this—likely due to the difficulty in proving causation. The Court of Appeal has since confirmed that a pregnant person cannot be found guilty of any harm short of death (the Offences Against the Person) caused by behaviour during pregnancy.^172^ Lord Dyson stipulated that ‘the court should be slow to interpret general criminal legislation as applying’ to fetuses.^173^ Importantly, civil law has, to date, conclusively ruled out any possibility of claims brought by a child based on a pregnant person’s behaviour.^174^ Similarly, in *Re F*, the Court of Appeal delineated the acceptable limits of legal intervention aimed at controlling behaviour during pregnancy for the benefit of the fetus, with the Court thwarting any attempted use of wardship to protect the fetus.^175^ Despite law’s insistence that fetuses have no legal standing and pregnant people cannot be “policed”, pregnant people continue to face considerable medical and social pressure to conform to lifestyle restrictions during pregnancy. These include, inter alia, considerable social shaming for drinking during visible pregnancy,^176^ mandatory carbon monoxide testing policies,^177^ or recording prenatal alcohol usage,^178^ aiming to coerce pregnant people into making particular choices.^179^

At the same time, legislation and policy favour a pregnant person’s autonomy and active involvement in birth decision-making—including the right to decline recommended treatment. A central tenet of modern obstetric care is choice and this is reflected in current NICE guidelines recognising choice about place of birth as being important for the pregnant person’s health and wellbeing.^180^ This choice includes freebirthing—the active decision to birth without trained healthcare providers present—despite available obstetric care.^181^ However, where birth choices fall outside of sociocultural norms, as freebirthing often does, law often recognises fetuses’ potential need for protection *from* the pregnant person, notwithstanding the remoteness of risk, as explained further below. Furthermore, due to their condition and supposed temperamentality, pregnant people are subject to intense medical gaze. With birth choices, especially maternal request caesarean sections,^182^ homebirthing,^183^ and freebirthing,^184^ regulatory measures are conceptualised as necessary to minimise potential harm to the fetus. Notwithstanding this, a capacitous pregnant person is afforded the absolute right to refuse a caesarean section, even if this will be harmful to the fetus.^185^ The putative father, medical professionals, and courts cannot compel the procedure.^186^ However, this oft-repeated principle has not precluded legal mechanisms manipulating pregnant people’s compulsion and compliance.^187^

Obstetric advice is preoccupied with fetal outcomes, and obstetricians are trained and enabled by technology to see the fetus as a ‘second patient’,^188^ as explained above. Ethical dilemmas are thought to arise due to conflicting interests or choices of pregnant persons and fetuses,^189^ for example, where it is thought that a caesarean delivery might better protect fetal health, but the pregnant person does not want to have a surgical delivery. In such instances, law becomes involved, usually where the pregnant person is unwilling to compromise on their birth choice; thereby, prioritising their *own* welfare, rather than the fetus’, which is contrary to what is expected of them by healthcare providers. Case law has revealed the extent to which ‘good motherhood’ underpins these mechanisms, and this is demonstrated through routine judicial descriptions of pregnant people appearing on matters related to their antenatal care as “mothers”. As discussed below, in many cases, pregnant people are explicitly called ‘mother’^190^ or ‘expectant mother’.^191^

When considering the deployment of maternal language, it is easy to see how “good motherhood” narratives are explicit in many of the (en)forced caesarean cases. As the pregnant person is described as a mother, there is often the determination that it is in the incapacitated pregnant person’s ‘best interests’ to deliver via caesarean, as it would ensure that a healthy baby is born.^192^ For example, in *DD*, Cobb J declared that

it must be in the best interests of any woman carrying a full-term child whom she wants to be born alive and healthy that such a result if possible should be achieved.^193^

In other cases, the view that pregnant people should always be willing to conform to medical advice—including potentially traumatic interventions—to protect a fetus, or risk inevitable guilt and failing as a ‘mother’, has been explicitly endorsed. In *Re P*, although obstetric intervention on an incapacitated pregnant person was not justifiable *solely* to secure the fetus’ survival, Jackson J posited that fetal welfare must be considered because of the ‘extremely adverse effect on [the respondent] if unnecessarily her child was not born safely’.^194^ Through the use of the word ‘unnecessarily’, Jackson J implied that P’s birth choices, which may have been the result of lived experiences and personal conceptions of welfare, were unnecessary when “good motherhood” was at stake. Jackson J did refer to the seriousness of the procedure, observing that a caesarean without consent is an ‘intervention of a very serious kind. It involves the need for restraint and sedation’.^195^ However, this concern was not related directly to P’s experience, and was ultimately swiftly dismissed when it is shortly thereafter concluded that ‘there is no doubt at all that it would be in the best interests of Mrs P for her baby to be safely delivered’^196^ without any explicit weighing of this against the severity of the intervention as described. The best interests analysis that is often deployed in the ‘forced caesarean’ cases is built from

[t]he cultural script of the foetus as vulnerable citizen and the idealization of good motherhood as selflessness produce prevailing discourses of putting the foetus first … Pregnant women, as mothers-to-be, need to demonstrate their commitment to idealized motherhood by following biomedical regimes of advice and surveillance.^197^

When we consider the Court of Protection’s (CoP) involvement in homebirthing, the extent of the legal blurring between fetus and baby is revealed. Such blurring is inconsistent with the lack of recognition of fetal personhood in English law, as noted. The language used in these cases further entrenches presumptions about pregnant people’s behaviour needing to be ‘self-sacrificial’ and altruistic. “Good motherhood” *qua* judicial value facilitates erasure of the pregnant person’s identity, reinforcing ideological commitment to maternal sacrifice, as discussed earlier in Section III(C). “Good motherhood” narratives underpin the actions of those surrounding pregnant people who assume that fetal welfare should be prioritised, even if this is at the expense of their own welfare, and this is based on the flawed conflation of gestation as motherhood. Rhetoric that identifies the pregnant person as a “mother” in judicial discourse has significant normative implications in affording priority to the fetal outcomes. The application of the capacity test and best interests test in the caesarean cases illustrates that where pregnant people seek to make decisions about their birth that are misaligned with ‘good motherhood’ and this comes before this court they are unlikely to have their wishes respected. Specifically, behaving in unanticipated ways while pregnant, and particularly not acting in accordance with medical advice is often taken as evidence that the pregnant person does not have decision-making capacity.^198^ Following this, and as outlined, judgments are quick to jump to the assumption that a healthy baby, above all considerations, is always in the best interests of individuals.^199^ The following cases demonstrate how judicial subscription to the notion of “good motherhood” *qua* value, and their discussion of pregnant people as mothers, guides particular judgments.^200^

The respondent in *East Lancashire Hospitals NHS Trust v GH*^201^ suffered from anxiety, depression, and acute agoraphobia. She had not attended any antenatal care outside her home and had gone into labour at home. Her labour had become obstructed and required urgent in-patient hospital obstetric treatment, and possibly an emergency caesarean section. Her refusal to comply resulted in CoP involvement. While she was referred to in the judgment by her initials (which was important to preserve her anonymity), MacDonald J referred to the fetus as “unborn baby”. In so doing, the language of the judgment collapses the supposedly clear legal distinction between a fetus and a baby. The term “unborn baby” illuminates the predisposition to view a fetus *as* a baby (even if “unborn”) and therefore a pregnant person as a mother (though still pregnant). This choice of language engages the particular sensibilities of CoP judges. Many have been judges in the family law courts, and so are ‘dedicated to upholding child welfare. It is simply unrealistic to suppose that the preservation of each life’ will not be a concern.^202^ Descriptions of a fetus as an “unborn baby” invoke the imagery of a “baby”, an existing and situated individual with particular vulnerabilities that require intervention and thus, we argue, make judgments centring fetal welfare much more likely. This decision in this case, ultimately that GH should be transferred to hospital and sedated in the process if necessary,^203^ demonstrates the extent to which adherence to “good motherhood” *qua* value guides judges to a particular outcome, prioritising the fetus over pregnant person. GH was described as not engaging in a decision-making process by not recognising serious risks to herself and her fetus,^204^ however, as one of the authors has argued elsewhere, GH did have a clear decision-making process and rationale—it was just not one that centred medical risks and fetal outcomes in the way a “good mother” would observe, as anticipated by a medicalised model of pregnancy.^205^

*A NHS Foundation Trust v An Expectant Mother* is a unique case in which it was said that the respondent was ‘[a] pregnant woman whose care is at the heart of this case [and was] *already* a mother, even though she [had] not yet given birth’.^206^ The respondent was diagnosed with severe agoraphobia, and was, therefore, presumed to lack capacity to choose the place of birth. Despite her stated freebirth preference, Holman J presumed that ‘but for her agoraphobia, the mother herself would opt for a hospital birth, as encouraged by her mother and partner’.^207^ Her choice to freebirth was trivialised, rendered a product of her agoraphobia, and ‘not the kind of wish that should be given legal weight’.^208^ Holman J tritely assumed that the respondent ‘dearly [wished] to give birth to a healthy baby, undamaged by the process of birth’,^209^ but was seemingly unconcerned by the potential damage to her by the birth. Determined to avoid a stillbirth at all costs, and without any evidence indicating that ‘she may not have an uneventful, spontaneous labour and vaginal delivery’,^210^ Holman J ignored any potential risks to the respondent related to induction or caesarean section. This was despite the fact that psychiatrists agreed that the use of force could risk psychological morbidity.

Regardless, the legal interest in fetal life was transformed into the respondent’s desire for a healthy baby and was enforced against her wishes.^211^ Holman J’s persistent use of the term “mother”, despite the lack of a parentable entity, further reinforced the prioritisation of fetal welfare and the expectations that the respondent should act according to “good motherhood” narratives. The judgment was based entirely around the pregnant person *qua* mother with mothering obligations, and the intent to mother. This was an easy conceptualisation due to the repeated insistence on referring to the respondent and fetus as “mother” and “expected baby”, respectively. The putative father in this case was referred to as the ‘mother’s’ “partner”’^212^; thereby, reaffirming that fatherhood is attributed based on the relationship with a mother and, even more notably, the different construction of “mothering” as (illogically) existing before birth.

Beyond judicial discourse, referring to pregnant people as mothers does not necessarily correspond to their own preferences. Research conducted in England specifically exploring antenatal clinic attendees’ terminology preferences, revealed a preference for “patient” in medical literature and contexts, rather than “mum” or other maternal rhetoric, and, if given the choice, being referred to by their name.^213^ While it is important to acknowledge this study’s limitations (it had a small sample size and was conducted over 25 years ago), doing so simply reiterates the need for more empirical work on contemporary preferences. Nevertheless, there are normative reasons that suggest the need to change the terminology employed. Within clinical settings, referring to pregnant people as patients acknowledges their relationship with health professionals, and describes the interaction when a person ‘is presented to an obstetrician (or other healthcare provider) and there exist forms of clinical management that are reliably predicted to result in net clinical benefit for them’.^214^ Cognisant that the use of the term “patient” could promote the pathologisation and (over)medicalisation of pregnancy,^215^ referring to pregnant people as mothers has, we suggest, a greater impact on autonomy than patients, because it imbues healthcare interactions with ‘good motherhood’ expectations. While mothers are expected to factor in their children’s welfare when making decisions, patients are theoretically empowered to make personal decisions about their health, factoring in subjective relevant considerations, and refusing treatment, even where it appears irrational, and regardless of the impact on others. This is, at least, the position that law reiterates in the leading authorities on the centrality of autonomy and consent.^216^ We recognise that there may be reason to question the centrality of an atomistic conception of autonomy in health law,^217^ and that this conception of patient autonomy may in fact contribute to the power structures that consistently enable legal mechanisms to place limitations on pregnant people. However, we do not have space to engage in a specific discussion of the value of other frameworks, for example, relational autonomy or ethics of care. Here, we only seek to observe that within the current paradigm, when interacting with healthcare professionals, a pregnant person is entitled to the same respect and treatment as others interacting with these professions.

Obstetrics is, perhaps, the only branch of medicine where a person is not, by default, referred to by their name but by their (potential) *future* social role as “mother”. The language used in healthcare interactions should promote autonomy and reflect obstetric care’s focus on pregnant people and their preferences. Furthermore, pregnant people should be afforded appropriate respect and treated as individuals, regardless of their intention to “mother” any resulting baby. They should not, however, be subsumed into performing that role by virtue of carrying a pregnancy. Critically, pregnant people remain, first and foremost, autonomous individuals.

## V. FUTURE DIRECTIONS

So far, we have illustrated the weakness of the conceptual link conflating gestation and mothering, and its harm to pregnant people, and to women in general. Based on Kingma’s account of the metaphysics of pregnancy, through the parthood model we have demonstrated the flaws in legal assumptions about mothering as flowing from gestation. We have argued for better recognition of the fact that gestation and parenting need not be innately related. While it is outside our scope in this article to outline all the necessary reforms to better respect pregnant people’s preferences, we will consider some of the avenues that we believe require some reform. Such reforms, in need of further research as to their exact nature, may collectively break the flawed and legally entrenched conceptual conflation of gestation and mothering. This is important to better reflect metaphysical facts, to ensure that pregnant people are appropriately recognised as the *only* patients throughout pregnancy, and to deconstruct “good motherhood” *qua* value within judicial decisions. Continued recognition of the established legal orthodoxy that a fetus is not a legal person is clearly insufficient, as pregnant people are *still* being coerced into obstetric intervention through legal processes,^218^ and are simultaneously subjected to social coercion too. The ‘precautionary approach’ to pregnancy has come to dominate prenatal care in the UK in the 21^st^ century: effectively uncertainty about risk of a behaviour is used as justification to encourage abstinence from that behaviour.^219^ The most infamous example being ‘low level drinking’—despite there being a lack of evidence that this is harmful, abstinence is encouraged on the grounds of ‘better safe than sorry’.^220^ Through a ‘precautionary approach’ to fetal welfare, pregnant people and people with the potential to become pregnant, are constantly socially and medically pressured into unnecessarily adapting their behaviours.^221^ Reminiscent of our discussion of how women are framed as potential mothers in Section III, above, in July 2021, in the first draft of their Global Alcohol Action Plan, the World Health Organization advocated that women of ‘childbearing age’ refrain from drinking due to their potential to become pregnant.^222^ Thankfully, this recommendation was removed in the second draft.^223^ Clearly, we are in need of wholesale reform across law, medical guidance, and in practice, to change the framing of pregnancy as parenting.

Various suggestions, taken together, will begin the deconstruction of the flawed conceptual assumption that gestation is equal to mothering. We consider three possibilities here: intention-based parenthood and changing the statutory definition of motherhood in the HFE Act 2008, decriminalising the offence of concealing a birth, and rethinking the language used during pregnancy.

### A. Intention-Based Parenthood

As others,^224^ and one of us elsewhere,^225^ have argued, one place to start reform is basing motherhood on intention rather than gestation. Intention-based parenthood recognises legal parental rights based on the relational role played to the child, rather than on biology. This approach is consistent with the need to legally recognise a person’s status as parent *ab initio*; namely, responsibility for the child and their welfare.^226^ A “parent” is thus defined in relation to their current (or potential) role in a child’s life, which deserves appropriate legal recognition. Attributing this status to the correct individual(s) is important because ‘legal parenthood is “a question of most fundamental gravity and importance”’.^227^ Automatic recognition of parenthood arising from gestation does not adequately capture the realities of the decision to parent, and the lived realities of persons who intend to parent but cannot gestate. Importantly, an intention-based model better caters to the wider variety of lived experiences of pregnancy, compared to the current matrifocal model based on gestation. An intention-based model would assign legal parenthood based on the intention to conceive a child and fulfil a parental role, as demonstrated by, for example, the HFEA legal parenthood consent forms following ART.^228^ An intention model could bridge the gap between legal parenthood and social parenthood, recognising the diversity in family forms.

We are cognisant of the potential concerns with an intention-based model but raise it as a matter for further investigation rather than advocating it as the solution to the problems outlined throughout this article. In what follows we address some of the key criticisms that might be raised. Intention-based parenthood has been criticised as an overtly

*male* approach to parenthood because it fits far more closely to men’s experience of procreation than to that of most women … for men, *all* women who carry children are surrogates. Relying upon their intention to produce and raise a child is a very convenient way for men to assert their parentage over children.^229^

The central claim here is essentially that focussing on intention potentially displaces the person undertaking gestational labour, with their body effectively being captured for reproduction. This concern is rooted in the worry that any person intending to become a parent could seek out a surrogate and ‘claim the “product” of these people’s labours when the child was born’^230^ and thus contributing to the commodification of female-assigned physiology. Such an argument holds an intention-based model causes more harm to pregnant people *overall* than the specific harms we have described in this article. However, such a position neglects the consideration that many pregnancies carried to term willingly—and not due to necessity^231^—are accompanied by the gestating individual’s specific *intention* to become a parent. Recognition of their intention would not harm these individuals. An intention-based parenthood model does not necessarily displace the gestating individual’s central role in the creation of a new human entity. We see no reason why an intention-based model would not start from the gestating person’s intention. The various hierarchies around intention require further investigation and explanation; for example, should the gestating person’s intention carry more weight, and how would this be recognised? Furthermore, this criticism of the intention-based model does not consider how innately locating motherhood in gestation is already causing significant harm in co-opting female-assigned physiology in the kind of biological determinism we have described; that dictates that people should mother entities that they birth (limiting female equality) and how people should behave during pregnancy (limiting bodily autonomy).

The intention-based model is also criticised for creeping ‘closer to characterising children more openly as a form of property which can be transferred to others’.^232^ This argument finds its foothold in the belief that parenthood is a status-based institution, rather than one that maybe contracted out. It follows that intention-based parenthood approach provides IPs with property rights in another person, the surrogate-born baby,^233^ thereby demeaning the baby’s personhood. These arguments fail to recognise that fetuses cannot be the subject of rights in the first place, as we have argued, and fails to recognise the alignment of the intentions with the best interests of resulting children, as seen in parental orders following surrogacy. Moreover, if recording parental intentions innately demeans children to property, care arrangements could be similarly criticised.

While we believe these concerns deserve to be properly addressed, but there is still merit in an intention-based model. Separating motherhood from gestation will contribute to the de-gendering of legal motherhood and ensure that those able to gestate are not unduly burdened with automatic parental responsibility. Without gestation’s definitive link to motherhood, trans/masculine birth parents similar to *McConnell*,^234^ would be recognised properly as legal fathers, which would reflect their intentions and lived reality. Recognising intention as the important factor in reproduction and the co-produced relationships surrounding reproduction better supports modern families. For example, enabling more than two people’s recognition as legal parents, irrespective of sex or gender,^235^ specifically in that they intend to parent and this recognition is in the child’s best interests.

An intention-based parenthood model is a huge reform to a fundamental principle of English law. It has been suggested for some time and no action has resulted.^236^ Consequently, it is worth noting that some smaller reforms that enable aspects of intention-based parenthood to be realised could still bring some benefits. An example of such a reform would be allowing gestators/legal mothers to abdicate parental responsibility earlier than six weeks post-birth, recognising their intentions not to parent, such as in surrogacy.^237^ Given the weighty responsibility attached to legal motherhood, we believe an intentional component is necessary. Greater recognition of parenting’s social aspects betters supports the child’s best interests, assigning legal responsibility to the appropriate individual(s),^238^ that is, the “social” parents. This would allow those ‘parenting’ to make the necessary arrangements related to the day-to-day care of a child, recognising they are best placed to make decisions reflective of their welfare. In surrogacy, intended parents would be recognised as legal parents from birth, reflecting the lived reality of these arrangements.^239^

### B. Decriminalisation

Criminal reform is also needed, and we echo calls to repeal the offence of concealment of birth, as we explained it perpetuates a gendered injustice relating to mothering expectations.^240^ This reform offers better support for individuals experiencing a difficult pregnancy or who find themselves in difficult circumstances because of a specific vulnerability (e.g., a victim of domestic abuse). Repeal of this offence and those related further involves removing the requirement that the birthing person be identifiable against their wishes. Furthermore, while we have not discussed abortion in detail due to space limitations, decriminalisation of abortion serves to sever the link between gestation and mothering to the protection of all (including those who are carrying wanted pregnancies). Notions of “good motherhood” underpin the Abortion Act 1967, with motherhood still assumed to be ‘women’s destiny’, and opt-out only based on unsuitability,^241^ rather than choice, feeding into the construction of pregnant people *as* mothers. We have demonstrated this construction has a significant impact on the way people are treated and their available choices throughout pregnancy.

### C. Language

Finally, significant effort in reforming the language used during pregnancy is needed. Armstrong notes how pregnancy has been rhetorically constructed as separate and independent from the person, seen in the shift of ‘pregnant’ as something a person ‘is’ to ‘regarding pregnancy as something carried’.^242^

Careful consideration of legislative drafting is necessary,^243^ for example, there is a need to ensure language does not conflate pregnancy and mothering either implicitly or explicitly (as in the HFE Act 2008). Our focus here, however, is on the routinisation of maternal rhetoric in obstetric care. Healthcare providers’ language should support autonomy and reflect the pregnant person as receiving care. However, many healthcare providers continue to use maternal rhetoric as terms of endearment and accepted practice.^244^ Such rhetoric perpetuates expectations felt by pregnant people regarding decisions about their privacy and welfare. This happens in subtle but pervasive ways as ‘language has a powerful influence over the way human beings think’.^245^ Referring to a pregnant person as a mother when recommending lifestyle advice prevents them from questioning or challenging the advice, without feeling like they are somehow failing in their parenting project. This maternal rhetoric overly focuses on the value of pregnancy as making a parent, minimising the experience of pregnancy as a physiological transformation. Equally, it can be distressing for a pregnant person who does not intend to mother to have to explain this to a new healthcare provider at every interaction. Going over choosing adoption or being a surrogate repeatedly is tiring and draining, and may result in feeling of shame or uncertainty regarding their decisions.^246^ Finally, there are significant harms for pregnant trans/masuline and non-binary people who may not want to be considered mothers; the term may unnecessarily heighten the dysphoria potentially engendered by pregnancy. Although some pregnant people may prefer maternal terms, the harm of its consistent and accepted deployment *as standard* outweighs this preference. We maintain that, as default, pregnant people should be referred to by their preferred name in healthcare settings—like other patients—and that this would promote their autonomy and encourage appropriate healthcare decision-making by reinforcing their status as individuals rather than their perceived (potential) social role. Additionally, medical literature should refer to pregnant people as such (or even “pregnant patients” depending on context) rather than “expectant mothers”, as this accurately describes their position within the healthcare context.

We suggest that respectful obstetric care encompasses ‘a manner that maintains their dignity, privacy and confidentiality, ensures freedom from harm and mistreatment, and enables informed choice and continuous support during labour and childbirth’.^247^ An important approach within this is good communication, encompassing appropriate terms of address for pregnant people—by default by name and, upon request, by other terms. Training and adequate policy reform aids, and further contributes to tackling the aforementioned persistent gendered discrimination.

We appreciate that language changes within clinical contexts are insufficient to address all the current problems in obstetric care with regards to limited choice; however, fundamentally, the construction of the pregnant person and fetus *must* change, as this as at the root of many issues. Additionally, ‘[b]ecause language can be a catalyst for changing the way doctors think or approach patient care’^248^ the shift away from “mother” in antenatal care might fuel broader change, and importantly, improve the experience for some individuals. The introduction of more resources supporting pregnant people’s decision-making, such as about place of birth, will always be limited in terms of results without a broader shift away from “good motherhood” narratives that are routinely enabled by language use that drive and underpin healthcare providers’ expectations.

## VI. CONCLUSION

In this article, we have demonstrated law’s conceptual conflation between gestation and mothering and its harmful consequences for pregnant people. Using Kingma’s account of the metaphysics of pregnancy and the parthood model, we have argued that gestation, although a unique form of reproductive labour, is not “mothering”. Furthermore, we have suggested that “good motherhood” narratives impact pregnant people’s available choices during and after pregnancy. Echoing de Crespigny, we contend that the use of correct terminology describing pregnant people

is important in assisting decision making for pregnant women, such decisions sometimes being taken at times of great stress. The terminology used also indicates that the medical practitioner understands and respects the different ethical and legal positions of the fetus and baby, pregnant woman and mother.^249^

We have explained how and why gestation’s conflation with mothering perpetuates specific harms to gestating individuals not intending to parent (for example, surrogates), gestating individuals not identifying as women, and pregnant people making decisions about their birthing preferences that fall outside of sociocultural norms. Taken alongside other criticisms of how pregnant people are assumed to be legal mothers, our arguments contribute to the existing literature on parenthood and gestation by demonstrating the inaccuracies of the assumptions underpinning legal motherhood and its attribution to pregnant people. The assumption that mothering flows from gestation (based on the flawed assumption that gestation *is* a form of mothering) is not logical but is legally and socio-culturally represented.

To conclude, the inadequate differentiation between gestation and motherhood (and mothering) in law continues to result in the inappropriate and premature attribution of parenting responsibilities during pregnancy and after birth to gestating individuals. We have illustrated in this article some of the harms that result and some of the potential reforms that would go some way to recognising that pregnancy and mothering are distinct. Further consideration of the perpetuation of gestation *qua* mothering and how the resulting harms may be addressed and remedied is important to reflect further on the best possible solutions.

## Footnotes

1 Human Fertilisation and Embryology Act 2008 (HFE Act 2008), s 33(1).

2 Children Act 1989, ss 2(1), 2 (2)(a).

3
*Ampthill Peerage Case* [1977] AC 547, per Lord Simon o’Glaisdale at 577.

4 K O’Donovan, ‘Constructions of Maternity and Motherhood in Stories of Lost Children’ in Jo Bridgeman and Daniel Monk (eds), *Feminist Perspectives on Child Law* (Routledge-Cavendish 2000) 73.

5 Emphasis added.

6 We use the term ‘pregnant person’ to be inclusive of people who carry pregnancies not identifying as women. Overwhelmingly, people with the physiology to gestate identify as women, with this playing a substantial role in the historical subordination of pregnant people. Inclusive terminology is crucial to prevent the erasure of minorities struggling to access adequate reproductive healthcare.

7 S Halliday ‘Protecting Human Dignity: Reframing the Abortion Debate to Respect the Dignity of Choice and Life’ (2016) 13 J Contemp Issues Law 287, 287.

8 E Kingma, ‘Were You a Part of Your Mother?’ (2019) 128 Mind 609, 626.

9 ibid; E Kingma, ‘Lady Parts: The Metaphysics of Pregnancy’ (2018) 82 Roy I Ph S 165; E Kingma and S Finn, ‘Neonatal Incubator or Artificial Womb? Distinguishing Ectogestation and Ectogenesis using the Metaphysics of Pregnancy’ (2020) 34 Bioethics 354.

10 Kingma (n 8).

11 P Singh, ‘Fetuses, Newborns & Parental Responsibility’ (2020) 46 J Med Ethics 188, 190.

12 BP Blackshaw and D Rodger, ‘Parental Responsibilities and Moral Status’ (2021) 47 J Med Ethics 187.

13 RP Petchesky, ‘Fetal Images: The Power of Visual Culture in the Politics of Reproduction’ (1987) 13 Feminist Stud 263.

14 Kingma (n 8) 626.

15 ibid 624.

16 C Wells, ‘On the Outside Looking In: Perspectives on Enforced Caesareans’ in S Sheldon and M Thomson (eds), *Feminist Perspectives on Health Care Law* (Cavendish Publishing Ltd 1998) 237, 255.

17 ibid.

18 BK Rothman, *The Tentative Pregnancy: Prenatal Diagnosis and the Future of Motherhood* (Penguin Books 1986) 114; Petchesky (n 13) 273; EC Romanis and others, ‘Reviewing the Womb’ (2021) 47 J Med Ethics 820.

19 Kingma (n 8) 634–35.

20 K Greasley, *Arguments about Abortion: Personhood, Morality, and Law* (OUP 2017); EC Romanis ‘Challenging the “Born Alive” Threshold: Fetal Surgery, Artificial Wombs, and the English Approach to Legal Personhood’ (2020) 28 Med L Rev 93.

21 HT Engelhardt, *The Foundations of Bioethics* (OUP 1986) 111; EC Romanis, ‘Is “viability” viable? Abortion, Conceptual Confusion and the Law in England and Wales and the United States’ (2020) 7 J Law Biosci 21. https://doi.org/10.1093/jlb/lsaa059.

22 We want to be clear that we are not suggesting continuing a pregnancy is a form of self-care, just that it can be where a person wants to become a parent. Equally, for a person not wanting to become a biological parent, abortion would be a form of self-care.

23 A Stone, *Being Born: Birth and Philosophy* (OUP 2019) 38.

24
*Re G (Children)* [2006] UKHL 43, per Baroness Hale at [34].

25 Singh (n 11) 190.

26 Stone (n 23) 3.

27 ibid.

28 ibid 5.

29 Children and Young Persons Act 1933, s1(1) as amended by Serious Crime Act 2015, s 66.

30 H Watt, *The Ethics of Pregnancy, Abortion and Childbirth: Exploring Moral Choices in Childbearing* (Routledge 2016) 126.

31 Blackshaw and Rodger (n 12) 188.

32 P Williams, *The Alchemy of Race and Rights* (Virago Press 1993) 184.

33 M Power and J Schulkin, *The Evolution of the Human Placenta* (John Hopkins University Press 2012) 1.

34 GS Dawe and others, ‘Cell Migration from Baby to Mother’ (2007) 1 Cell Adh Migr 19.

35 Involving the continued “making” an individual entity in shaping the child’s experiences.

36 A Smajdor, ‘The Moral Imperative for Ectogenesis’ (2007) 16 Camb Q Healthc 336, 342.

37 Z Mahmoud, ‘Surrogates across the Atlantic: Comparing the Impact of Legal and Health Regulatory Frameworks on Surrogates' Autonomy, Health, and Wellbeing’ (unpublished PhD thesis, University of Exeter 2022).

38 Blackshaw and Rodger (n 12) 187.

39 L de Crespigny and others, ‘Mothers and Babies, Pregnant Women and Fetuses’ (2005) 106 BJOG 1235, 1235.

40 A Brown, ‘Trans Parenthood and the Meaning of “Mother”, “Father” and “Parent”—*R (McConnell and YY) v Registrar General for England and Wales* [2020] EWCA Civ 559’ (2020) 29 Med L Rev 157.

41 Mahmoud (n 37).

42 M Besse and others, ‘Experiences with Achieving Pregnancy and Giving Birth Among Transgender Men: A Narrative Literature Review (2020) 93 Yale J Biol Med 517, 520.

43 N Borg Cunen and others, “‘A Small Person that we Made”—Parental Conceptualisation of the Unborn Child: A Constructivist Grounded Theory’ (2022) 104 Midwifery 103198.

44 EC Romanis, ‘Abortion & “Artificial Wombs”: Would “Artificial Womb” Technology Legally Empower Non-gestating Genetic Progenitors to Participate in Decisions about how to Terminate Pregnancy in England and Wales?’ (2021) 8 J Law Biosci 32. https://doi.org/10.1093/jlb/lsab011.

45 Children Act 1989, s 2(2).

46 A parental order is a post-birth order which transfers legal parenthood from the surrogate to the intended parent(s): HFE Act 2008 ss 54, 54A.

47 K Horsey, ‘Fraying at the Edges: UK Surrogacy Law in 2015’ (2016) 24 Med L Rev 608.

48 J McCandless, ‘Reforming Birth Registration Law in England and Wales?’ (2017) 4 RBMS 52, 54.

49 Mahmoud (n 37).

50 E.g., Surrogacy UK, ‘Surrogacy in the UK: Further Evidence for Reform. Second Report of the Surrogacy UK Working Group on Surrogacy Law Reform’ https://surrogacyuk.org/2018/12/31/surrogacy-survey-2018-results/ (last accessed 1 April 2022); Mahmoud (n 37).

51 Though it is important to note that funding is not equal and is available to cis-gendered heterosexual couples, where it is available at all. Furthermore, funding for IVF is far more limited than funding for intra-uterine insemination, which can also thwart the reproductive objectives of some same sex couples.

52 HFE Act 2008, ss 42-47.

53 Adoption and Children Act 2002 (ACA 2002) s 144.

54 HFE Act 2008 ss 36, 42; E Jackson, ‘What is a Parent?’ in Alison Diduck and Katherine O'Donovan (eds), *Feminist Perspectives on Family Law* (Routledge-Cavendish 2007) 59.

55 A Bainham, ‘Parentage, Parenthood and Parental Responsibility: Subtle, Elusive Yet Important Distinctions’ in A Bainham and others (eds), *What is a Parent? A Socio-Legal Analysis* (Hart Publishing 1999) 29.

56 We thank an anonymous reviewer for raising this nuance.

57
*Marckx v Belgium* ECHR 13 June 1979.

58 Bainham (n 55) 29.

59 Children Act 1989; K Horsey, ‘Legal Parenthood and Parental Responsibility’ in Ruth Lamont (ed) *Family Law* (2 edn, OUP 2022) 303.

60 A Rich, *Of Woman Born: Motherhood as Experience and Institution* (Norton 1986).

61
*Paton v British Pregnancy Advisory Service Trustees* [1979] QB 276; *C v S* [1987] 1 All ER 1230; *Attorney-General’s Reference No. 3 of 1994* [1997] 3 All ER 936; *Re MB (Medical* Treatment) [1997] 2 FLR 426 (CA) 444.

62 Romanis (n 20) 99.

63
*Re F (in utero)* [1988] Fam 22.

64 s 33(1), emphasis added.

65 HFE Act 2008 ss 35–41, 42–47; R D’Alton Harris, ‘Mater Semper Incertus Est: Who’s Your Mummy?’ (2014) 22 Med L Rev 357, 358–59.

66 E.g., the presumption that a husband is a father, and that a female partner can be named as a parent under HFE Act 2008 ss 35–41, 42–47.

67 MA Fineman, *The Neutered Mother, the Sexual Family and other Twentieth Century Tragedies* (Routledge 1995) 38.

68 LM Finley, ‘Breaking Women's Silence in Law: The Dilemma of the Gendered Nature of Legal Reasoning’ (1989) 64 Notre Dame L Rev 886, 894.

69 CE Schneider, ‘The Channelling Function in Family Law’ (1992) 20 Hofstra L Rev 495.

70 HFE Act 2008, s 35.

71 ibid s 37; HFEA PP Consent form, HFEA WP Consent form.

72 ACA 2002, s 111.

73 HFE Act 2008 s 42; HFEA PP Consent form, HFEA WP Consent form.

74 Children Act 1989, s 4.

75 Explanatory notes to the Welfare Reform Act 2009, s 30.

76 HFE Act 2008, s 54.

77 P Lowe, *Reproductive Health and Maternal Sacrifice* (Palgrave Macmillan 2016) 6–7.

78 S Firestone, *The Dialectic of Sex* (republication, Verso 2015) 65; M O’Brien, ‘The Dialectics of Reproduction’ in A O'Reilly (ed) *Maternal Theory: Essential Readings* (Demeter Press 2007) 49.

79 K O’Donovan and J Marshall, ‘After Birth: Decisions about Becoming a Mother’ in A Diduck and K O’Donovan (eds), *Feminist Perspectives on Family Law* (Routledge-Cavendish 2007) 103.

80 ‘The fundamental part that from the beginning of history doomed woman to domestic work and prevented her taking part in the shaping of the world was her enslavement to the generative function’: S de Beauvoir, *The Second Sex* (C Borde and S Malovany-Chevallier trs, Knopf 1949) 117.

81 JM Ussher, *The Psychology of the Female Body* (Routledge 1989) 80.

82 G Corea, *The Mother Machine: Reproductive Technologies from Artificial Insemination to Artificial Wombs* (Women’s Press 1988) 170.

83 RZ Eisenstein, *The Female Body and the Law* (University of California Press 1988) 80; DE Roberts, ‘Racism and Patriarchy in the Meaning of Motherhood’ (1993) 1 JGSPL 1, 10.

84 Romanis (n 44).

85 MJ Frug, ‘A Postmodern Feminist Legal Manifesto (An Unfinished Draft)’ (1992) 105 HLR 1045, 1059–60.

86 Horsey, ‘Fraying at the Edges’ (n 47); D’Alton Harris (n 65); K Horsey and S Sheldon, ‘Still Hazy After All These Years: The Law Regulating Surrogacy’ (2012) 20 Med L Rev 67, 67–69.

87 ACA 2002, s 52(3); HFE Act 2008, s 54(7)—no mechanism for dispensing with consent (even if in the child’s best interests); *Re AB (Surrogacy: Consent)* [2017] 2 FLR 217.

88 C Shalev, *Birth Power* (Yale University Press 1999) 121.

89 K O’Donovan, ‘Enfants Trouvés, Anonymous Mothers and Children’s Identity Rights’ in K O’Donovan and GR Rubin (eds), *Human Rights and Legal History: Essays in Honour of Brian Simpson* (OUP 2000) 66, 77.

90 E Milne, *Criminal Justice Responses to Maternal Filicide: Judging the Failed Mother* (Emerald Publishing 2021) 103.

91
*Ampthill Peerage Case* (n 3).

92 Family Law Reform Act 1969, s 27.

93 ibid ss 20–22.

94 E.g., where the father is unknown/whereabouts are unknown or the mother has reason to fear their safety if father is contacted in relation to the registration; BRD Act 1953, s 2B as amended by Welfare Reform Act 2009.

95 s 2a, as amended by the Welfare Reform Act 2009.

96 We do not have space to explore these in this article.

97 A Bainham, ‘What is the Point of Birth Registration?’ (2008) 20 CFLQ 449, 470.

98 We thank an anonymous reviewer for highlighting this.

99 BRD Act 1953, s 10.

100 D NeJaime, ‘The Nature of Parenthood’ (2017) 126 Yale LJ 2260, 2272.

101 HFE Act 2008, s 33(1).

102 ibid, ss 42–44.

103 A Brown, ‘*Re G; Re Z (Children: Sperm Donors: Leave to Apply for Children Act Orders)*: Essential Biological Fathers and Invisible Legal Parents’ (2014) 26 CFLQ 237, 237–51.

104
*R (McConnell and YY) v Registrar General* [2020] EWCA Civ 559.

105
*R (on the application of TT) v The Registrar General for England and Wales* [2019] EWHC 2384 Fam.

106 ibid [54], [133].

107
*McConnell* (n 104) [279]

108 ibid [35].

109 D’Alton Harris (n 65) 359.

110
*Re TT* (n 105) [137].

111 EC Romanis, ‘Regulating the “Brave New World”: Ethico-Legal Implications of the Quest for Partial Ectogenesis’ (PhD thesis, University of Manchester 2020) 231.

112 GRA 2004, s 12.

113 S Gilmore, ‘The Legal Status of Transsexual and Transgender Persons in England and Wales’ in Jens Scherpe (ed), *The Legal Status of Transsexual and Transgender Persons*, (CUP 2015).

114 L Davis, ‘Deconstructing Tradition: Trans Reproduction and the Need to Reform Birth Registration in England and Wales’ (2021) 22 Int J Transgender Health 179, 182.

115 Fineman (n 67).

116 CR Sunstein, ‘On the Expressive Function of Law’ (1996) 144 Univ PA L Rev 2021, 2022.

117
*Re TT* (n 105) [234], [243], [244].

118 C Fenton-Glynn, ‘Deconstructing Parenthood: What Makes a Mother?’ (2020) 79 CLJ 34.

119 We are grateful to Liam Davis for discussion on this point. See: E Higgs, ‘A Cuckoo in the Nest? The Origins of Civil Registration and State Medical Statistics in England and Wales’ (1996) 11 Contin Change 115.

120 HL Deb 12 December 2007, vol 697, col 299ff.

121 J McCandless and S Sheldon, ‘The Human Fertilisation and Embryology Act (2008) and the Tenacity of the Sexual Family Form’ (2010) 73 MLR 175, 194–96.

122 K Czapanskiy, ‘Volunteers and Draftees: The Struggle for Parental Equality’ (1991) 38 UCLA L Rev 1415, 1415–16.

123 We thank Sara Fovargue for raising this point. See: A Coe, ‘Dads Caring for Their Kids: It’s Parenting not Babysitting’ <https://www.theatlantic.com/sexes/archive/2013/01/dads-caring-for-their-kids-its-parenting-not-babysitting/267443/> accessed 4 July 2022.

124 N Dowd, ‘Rethinking Fatherhood’ (1996) 28 Fla L Rev 523, 523.

125 Offences Against the Person Act 1861, s 60.

126 ibid s 27.

127 E Milne, ‘Concealment of Birth: Time to Repeal a 200-Year-Old “Convenient Stop-Gap”?’ (2019) 27 Fem Leg Stud 139, 146.

128 ibid 140–54.

129 E Milne, ‘Putting the Fetus First–Legal Regulation, Motherhood, and Pregnancy’ (2020) 27 Mich J Gender & L 149, 154.

130 Fineman (n 67) 38.

131 K O’Donovan, *Family Law Matters* (Pluto Press 1993) 23.

132 SB Boyd, ‘Motherhood and Law: Constructing and Challenging Normativity’ in M Davies and V Munro (eds), *The Ashgate Companion to Feminist Legal Theory* (Ashgate 2013) 270.

133
*Re G* (n 24).

134 HFE Act 2008, s 33; A Brown, *What is the Family of Law? The Influence of the Nuclear Family* (Hart Publishing 2019) 115.

135
*Re G* (n 24).

136 J Herring and O Powell, ‘The Rise and Fall of Presumptions Surrounding the welfare Principle’ (2013) 43 Fam Law 553, 555.

137 Children Act 1989, s1(1).

138 Herring and O’Powell (n 136) 555; *Re G* (n 24) [3], [36].

139 Children Act 1989, s 2(2).

140
*Paton* (n 61); *C v S* (n 61).

141 EC Romanis, ‘Pregnant Women May Have Moral Obligations to Foetuses They Have Chosen to Carry to Term, But the Law Should Never Intervene in a Woman's Choices during Pregnancy’ (2017) 6 Manchester Rev L Crime & Ethics 69, 74.

142
*Re F* (n 63).

143 ibid 196.

144 L Purdy, ‘Are Pregnant Women Fetal Containers?’ (1990) 4 Bioethics 273.

145 In relation to homicide see *A-G’s Reference* (n 61); in relation to the right to refuse medical treatment see *Re MB* (n 61).

146
*Re G* (n 24) [36].

147 Blackshaw and Rodger (n 12) 188.

148 Emphasis added.

149 Drafting Techniques Group of the Office of the Parliamentary Counsel, Office of the Parliamentary Counsel: Drafting Guidance (22 June 2020) 2.1.

150 Ministerial and other Maternity Allowances Act 2021; Lords Amendments to the Ministerial and other Maternity Allowances Bill (25 February 2021); HC Deb 1 March 2021, vol 690, col 61.

151 Department of Health and Social Care, ‘Gamete (Egg, Sperm) and Embryo Storage Limits: Response to Consultation’, <https://www.gov.uk/government/consultations/egg-sperm-and-embryo-storage-limits/outcome/gamete-egg-sperm-and-embryo-storage-limits-response-to-consultation> last accessed 13 October 2021.

152 M Mukherjee and Z Mahmoud, ‘Adjudicating Existence: The Changing Socio-legal Landscape of Reproduction and Disabilities Alongside Prenatal Genetic Technologies’ (2023) Am J Soc *forthcoming*.

153 Rothman (n 18) 114.

154 Kingma (n 8); Romanis and others (n 18).

155 National Institute for Health and Clinical Excellence, Caesarean birth (NG192, 2021) 4.

156 National Institute for Health and Clinical Excellence, Abortion Care (NG140, 2019).

157 British Medical Association, *BMA Guide to Effective Communication* (BMA 20160368, 2016) 5.

158 MJ Casper, ‘At the Margins of Humanity: Fetal Positions in Science and Medicine’ (1994) 19 ST & HV 308, 312–13; RP Horgan and others, ‘Ascertaining Women’s Choice of Title During Pregnancy and Childbirth’ (2005) 98 Ir Med J 55, 56; Mahmoud (n 37).

159 Blackshaw and Rodger (n 12) 187.

160 Lowe (n 77) 131–33.

161 L de Crespigny, ‘Words Matter: Nomenclature and Communication in Perinatal Medicine’ in Asim Kurjak and Frank A. Chervenak (eds), *Textbook of Perinatal Medicine*, vol 1 (2nd edn, CRC Press, 2006) 159.

162 L de Crespigny, ‘What’s in a Name – Is the Pregnant Woman a Mother? Is the Fetus a Baby?’ (1996) 36 ANZJOG 435, 436.

163 de Crespigny and others (n 39) 1236.

164 C Sanger, ‘Infant Safe Haven Laws: Legislating in the Culture of Life’ (2006) 106 Colum L Rev 753, 812.

165 R Arkell, ‘Mandatory Recording of Pregnant Women’s Drinking is Just One More Attempt to Police our Bodies.’ *Huffington Post Online.* 2020; E Lee and others, ‘Beyond “the Choice to Drink” in a UK Guideline on FASD: the Precautionary Principle, Pregnancy Surveillance, and the Managed Woman’ (2022) 24 Health Risk Soc 1, 3–5.

166 EM Armstrong, *Conceiving Risk, Bearing Responsibility: Fetal Alcohol Syndrome and the Diagnosis of Moral Disorder* (Johns Hopkins Press 2003) 216.

167 Mukherjee and Mahmoud (n 152); Lee and others (n 165).

168 Legal standing is only assumed at birth, *Burton v Islington Health Authority* [1993] QB 204.

169
*A-G’s Reference* (n 61).

170 ibid.

171 M Brazier and E Cave, *Medicine, Patients and the Law* (6th edn, Manchester University Press 2016) 346.

172
*CP v CICA* [2014] EWCA Civ 1554.

173 ibid [65].

174 With the exception of driving: Congenital Disabilities (Civil Liability) Act 1976, s 1.

175
*Re F* (n 63).

176 B Thom and others, ‘Drinking in Pregnancy: Shifting Towards the “Precautionary Principle”’ in S MacGregor and B Thom (eds), *Risk and Substance Use: Framing Dangerous People and Dangerous Places* (Routledge 2020).; Lee and others (n 165).

177 C Bowden, ‘Are We Justified in Introducing Carbon Monoxide Testing to Encourage Smoking Cessation in Pregnant Women?’ (2019) 27 Health Care Anal 128.

178 National Institute for Health and Clinical Excellence, Fetal alcohol spectrum disorder (QS204, 2022). Note that following consultation these proposals have been scrapped.

179 J Davidson, ‘Pregnant Pauses: Agoraphobic embodiment and the limits of (im)pregnability’ (2001) 8 Gend Plac Cult 283, 289.

180 National Institute for Health and Clinical Excellence, *Intrapartum care for healthy women and babies* (CG190, 2014) 1.1.

181 C Feeley and G Thomson, ‘Tensions and Conflicts in “Choice”: Women’s Experiences of Freebirthing in the UK’ (2016) 41 Midwifery 16, 17.

182 EC Romanis, ‘Why the Elective Caesarean Lottery is Ethically Impermissible’ (2019) 27 Health Care Anal 249.

183 A Nelson and EC Romanis, ‘The Medicalisation of Childbirth and Access to homebirth in the UK: Covid-19 and Beyond’ (2021) 29 Med L Rev 661.

184 ibid.

185
*Re MB* (n 61); *CP* (n 172) [66].

186
*St George’s Healthcare NHS Trust v S* [1998] 3 All ER 673.

187 Determination of incapacity in these cases has been subject to significant academic critique; S Fovargue and J Miola, ‘Policing Pregnancy: Implications of the Attorney-General’s Reference (No. 3 of 1994) (1998) 6 Med L Rev 265; S Halliday, *Autonomy and Pregnancy: A Comparative Analysis of Compelled Obstetric Intervention*, (Routledge 2016); Nelson and Romanis (n 183).

188 Halliday (n 187) 167.

189 Romanis and others (n 18) 283.

190
*A NHS Foundation Trust v An Expectant Mother* [2021] EWCOP 33.

191 ibid.

192 S Fovargue, ‘In whose Best Interests?: Childbirth Choices and Other Health Decisions’ (2021) 137 LQR 604.

193
*The Mental Health Trust, The Acute Trust, The Council v DD* [2014] EWCOP 11 [97].

194
*Re P* [2013] EWHC 4581 [17].

195 ibid [15].

196 ibid [17].

197 Lowe (n 77) 132.

198 S Halliday, ‘Court-Authorised Obstetric Intervention Insight and Capacity, a Tale of Loss’ in C Pickles and J Herring (eds), *Childbirth, Vulnerability and Law Exploring Issues of Violence and Control* (Routledge 2019) 179.

199 Fovargue (n 192).

200 On the impact and entrenchment of underlying legal values in judgments see: Timothy J Dodsworth, *The Underlying Values of German and English Contract Law* (CUP forthcoming).

201 [2021] EWCOP 18.

202 Thorpe LJ, ‘The Caesarean Section Debate’ [1997] 27 Fam L 663, 663.

203
*GH* (n 201) [40].

204 ibid [31].

205 Nelson and Romanis (n 183) 677-78.

206 R Fletcher, ‘On care, coercion and childbirth in the Court of Protection’ (*Open Justice Court of Protection Process*, 5 July 2021) <https://openjusticecourtofprotection.org/2021/07/05/on-care-coercion-and-childbirth-in-the-court-of-protection/ > accessed 8 July 2021 (emphasis added).

207
*An Expectant Mother* (n 190) [22].

208 Fletcher (n 206).

209
*An Expectant Mother* (n 190) [28].

210 ibid [12].

211 Fletcher (n 206).

212
*An Expectant Mother* (n 190) [1].

213 N Barra and RJ Liliford, ‘Not Clients, not Consumers and Definitely not Maternants’ (1996) 64 Eur J Obstet Gyn RB 197, 199; DL Byrne, T Asmussen and JM Freeman, ‘Descriptive Terms for Women Attending Antenatal Clinics: Mother Knows Best?’ (2000) 107 Br J Obstet Gynaecol 1233; TF Baskett, ‘What Women Want: Don’t Call Us Clients, and We Prefer Female Doctors’ (2002) 24 JOGC 572; Horgan and others (n 158) 56.

214 LB McCullough and others, *Professional Ethics in Obstetrics and Gynaecology* (CUP 2019) 30.

215 R Mander and J Murphy-Lawless, *The Politics of Maternity* (Routledge 2013) 14; Nelson and Romanis (n 183).

216
*Montgomery v Lanarkshire Health Board* [2015] UKSC 11.

217 H Biggs, ‘Reproductive Autonomy and Regulation: Challenges to Feminism’ (2010) 18 Fem Leg Stud 299, 302.

218 S Fovargue and J Miola ‘Are We Still Policing Pregnancy’ in C Stanton and others (eds), *Pioneering Healthcare Law: Essays in Honour of Margaret Brazier* (Routledge 2016).

219 E Lee and others (n 165) 17.

220 ibid.

221 R Arkell, ‘NICE Draft Quality Standards on FASD: A precautionary approach gone too far?’ (Wrisk Blog, 2021) <https://wrisk.org/guest-blog/nice-draft-quality-standards-on-fasd-a-precautionary-approach-gone-too-far/> accessed 13 October 2021.

222 World Health Organization, ‘Global Alcohol Action Plan: First Draft’ (2021). < https://cdn.who.int/media/docs/default-source/alcohol/action-plan-on-alcohol_first-draft-final_formatted.pdf?sfvrsn=b690edb0_1&download=true> accessed 18 July 2022.

223 World Health Organization, ‘Global Alcohol Action Plan: Second Draft)’ (2021) <https://cdn.who.int/media/docs/default-source/alcohol/alcohol-action-plan/ada-action-plan-on-alcohol_second-draft.pdf?sfvrsn=a994ba7a_3&download=true> accessed 18 July 2022.

224 K Horsey, ‘Legally Recognising Intention: Parenthood in Surrogacy and Assisted Conception’ (PhD thesis, University of Kent 2003); Kirsty Horsey, ‘Challenging Presumptions: Legal Parenthood and Surrogacy Arrangements’ (2010) 22 CFLQ 449, 451; K Horsey and E Jackson, ‘Discrimination and Reform of the Human Fertilisation and Embryology Act 1990?’ (2022) MLR (*forthcoming)*.

225 Mahmoud (n 37).

226 Bainham (n 55).

227
*Re HFEA (Cases A, B, C, D, E, F and G)* [2015] EWHC 2602 Fam [3].

228 Human Fertilisation and Embryology Authority, *Code of Practice* (9th end, rev July 2022).

229 G Douglas, ‘The Intention to be a Parent and the Making of Mothers’ (1994) 57 MLR 636, 637-38 (emphasis in original).

230 ibid.

231 Assuming good access to abortion.

232 Douglas (n 229) 640.

233 D Dickenson, *Property in the Body: Feminist Perspectives* (CUP 2017) 69.

234
*McConnell* (n 104); Horsey and Jackson (n 224).

235 Douglas (n 229) 639.

236 Horsey (n 224).

237 Law Commission, *Building Families Through Surrogacy: A New Law* (Law Com No 244, 2019) at 8.27ff.

238 Horsey and Jackson (n 224).

239 Mahmoud (n 37).

240 Milne (n 90).

241 Sally Sheldon, *Beyond Control: Medical Power and Abortion Law* (Pluto Press 1997) 42.

242 Armstrong (n 166) 9.

243 Horsey and Jackson (n 224).

244 Mahmoud (n 37).

245 de Crespigny and others (n 161).

246 Mahmoud (n 37).

247 World Health Organization, ‘WHO recommendations: intrapartum care for a positive childbirth experience’ (2018) <https://apps.who.int/iris/bitstream/handle/10665/260178/9789241550215-eng.pdf> accessed 13 October 2021).

248 C Cox and Z Fritz, ‘Presenting Compliant: Use of Language that Disempowers Patients’ (2022) 377 BMJ e066720.

249 de Crespigny (n 162) 436.

## Notes

We are grateful to Zoe Tongue, Liam Davis, Dr Kirsty Horsey and Dr Emma Milne for their helpful comments on earlier drafts of this manuscript. Z.M. is supported by a Wellcome Trust Doctoral Studentship, based at the Wellcome Centre for Cultures and Environments of Health (Wellcome Trust Core Award Grant Number 203109/Z/16/Z).

